# Genome wide survey of G protein-coupled receptors in *Tetraodon nigroviridis*

**DOI:** 10.1186/1471-2148-5-41

**Published:** 2005-07-15

**Authors:** Raghu Prasad Rao Metpally, Ramanathan Sowdhamini

**Affiliations:** 1 National centre for biological sciences, Tata institute of fundamental research, UAS-GKVK campus, Bellary road, Bangalore 560065, India

## Abstract

**Background:**

The G-protein-coupled receptors (GPCRs) constitute one of the largest and most ancient superfamilies of membrane proteins. They play a central role in physiological processes affecting almost all aspects of the life cycle of an organism. Availability of the complete sets of putative members of a family from diverse species provides the basis for cross genome comparative studies.

**Results:**

We have defined the repertoire of GPCR superfamily of *Tetraodon *complement with the availability of complete sequence of the freshwater puffer fish *Tetraodon nigroviridis*. Almost all 466 *Tetraodon *GPCRs (Tnig-GPCRs) identified had a clear human homologue. 189 putative human and *Tetraodon *GPCR orthologous pairs could be identified. *Tetraodon *GPCRs are classified into five GRAFS families, by phylogenetic analysis, concurrent with human GPCR classification.

**Conclusion:**

Direct comparison of GPCRs in *Tetraodon *and human genomes displays a high level of orthology and supports large-scale gene duplications in *Tetraodon*. Examples of lineage specific gene expansions were also observed in opsin and odorant receptors. The human and *Tetraodon *GPCR sequences are analogous in terms of GPCR subfamilies but display disproportionate numbers of receptors at the subfamily level. The teleost genome with its expanded set of GPCRs provides additional and interesting comparators to study both evolution and function of these receptors.

## Background

The G-protein-coupled receptors (GPCRs) constitute one of the largest and most ancient superfamilies of membrane proteins, accounting for 1–2% of the vertebrate genome. GPCRs are characterized by the presence of highly conserved molecular architecture encoding seven transmembrane (TM) hydrophobic regions linked by three extracellular loops that alternate with three intracellular loops [1]. The extracellular N-terminus is usually glycosylated and the cytoplasmic C-terminus is generally phosphorylated. The extracellular side of these receptors contains residues that are specifically recognized by ligands and is therefore involved in ligand-specific binding. The endogenous ligands for GPCRs have exceptionally high chemical diversity. They include biogenic amines, glycoproteins, ions, lipids, nucleotides, peptides and proteases. Moreover, the sensation of exogenous stimuli such as light, odor and taste is also mediated via this superfamily of receptors. Ligand-induced activation of all GPCRs leads to a conformational change of the receptor and triggers a family of heterotrimeric GTP binding proteins (G proteins) and modulates several cellular signaling pathways.

GPCRs have been aggressively pursued as drug targets due to their central role in physiological processes affecting almost all aspects of the life cycle of an organism [2]. Almost half of the GPCRs are likely to encode sensory receptors and the rest of receptors could be considered as potential drug targets [3]. It is estimated that about 50% of all current drug targets are GPCRs and are the most successful of any target class in terms of therapeutic benefit [4,5]. A major goal of GPCR research is to expand the knowledge of GPCR structure/function in order to validate additional GPCR family members as tractable drug targets. Much effort, therefore, has been made to identify novel GPCRs and their ligands with potential therapeutic value [6-8].

The completion of several other vertebrate and invertebrate genome sequencing projects paves the way for "functional genomics". The quest for assigning function to putative gene products exploits the sequence and structural similarities to known genes and further could be elucidated using molecular biology techniques [9,10]. Such studies have important implications in biology and in understanding the evolution of distinct organisms. Sequencing of the model organisms can be an important source of information on the function of human target class members. For example, evolutionary comparison of GPCR sequences between species can help to identify conserved motifs and may recognize key functional residues [11-13]. The majority of GPCR functional data have been derived from studies in genetic models such as mice, rat, worm and *Drosophila*; additional species provide new comparators for GPCR studies. Teleost fish, *Tetraodon nigroviridis *is one of the smallest known vertebrate genomes. It has all the specialized functions of higher vertebrates and can be a good vertebrate model system to study [14,15]. The first available nearly complete sequence of *T. nigroviridis *genome now allows for the identification and analysis of its full set of GPCRs. Here, we describe the genome wide survey of Tnig-GPCR repertoire and a detailed analysis of opsin, fish-odorant receptors (FOR) and taste receptors (T1R).

## Results and discussion

**Figure 1:**
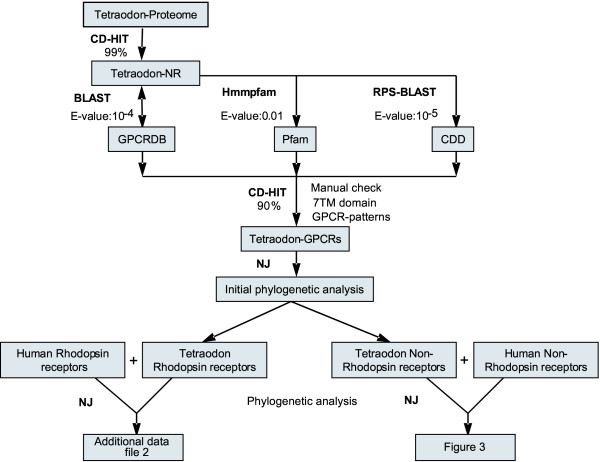
**Comprehensive approach for the identification and validation of Tnig-GPCRS**. All GPCR sequences from GPCRDB were compared against *Tetraodon *proteome database using BLASTP and hits were searched against GPCRDB using reverse BLAST. As complementary approaches, *Tetraodon *sequences were compared using Hmmpfam against Pfam and RPS-BLAST against CDD respectively. Finally, GPCR sequences are subjected to phylogenetic analysis as described in Methods.

Recent analysis of the genome sequence of the fresh water pufferfish *Tetraodon nigroviridis *genome (>90% sequence coverage) has shown that it possesses one of the smallest known vertebrate genomes and revealed a set of 27,918 predicted genes, much similar to the number of predicted genes in human genome [16,17]. In order to identify complete set of putative GPCRs within *Tetraodon *genome, we developed a comprehensive strategy (Figure 1). Table 1 summarizes 466 Tnig-GPCRs that were identified, out of which, to the best of our knowledge, 457 have not been reported before. The complete list of Tnig-GPCRs, including their sequence similarities to the functionally characterized GPCRs from human and other organisms, is available as Additional data file 1. GPCRs represent ~1.9% of total number of genes predicted from 340 mega base pair *T. nigroviridis *genome [14], which is comparable to those predicted in fly, mosquito and mammalian genomes [18]. Despite the higher sequence diversity of GPCRs in fly, mosquito, *C. elegans *and other vertebrates, sequence analysis suggests evolutionary conservation of GPCRs across phyla and that they might have ancient origins (data not shown). For almost all Tnig-GPCRs, a putative human GPCR homologue could be identified. 189 putative human and *Tetraodon *GPCR orthologous pairs are identified (see Additional data file 1).

**Table 1:** G protein-coupled receptors of *Tetraodon nigroviridis *(Tn). The numbers predicted in each family and sub-family are shown in comparison to humans (Hs)

**Receptor**	**Tn**	Hs*
**Glutamate-like**		
CASR like	9	1
GABA-B	4	2
Metabotropic glutamate	12	8
Taste1	4	4
Orphan	7	0
**Rhodopsin-like**		
Amine	71	40
Chemokine	42	42
Glycoprotein hormone/LGRs	5	8
MECA	24	22
Melatonin	3	3
Olfactory	22	460
Opsins	27	9
Peptide	88	60
Prostaglandin	12	15
Purine	48	42
Orphan	26	23
**Adhesion-like**		
BAI	3	3
CD97	1	1
CELSR	3	3
EMR	1	3
ETL	1	1
HE6	2	1
LEC	6	3
Orphan	12	9
**Frizzled-like**		
Frizzled	11	10
Smoothened	1	1
Taste2	0	13
**Secretin-like**		
CALCRL/CRHR	1+3 = 4	2+2 = 4
GLPR/GCGR	1+2 = 3	1+2+1 = 4
PTHR	3	2
GHRHR/PACAP/SCTR/VIPR	1+4+1+4 = 10	1+2+1+1 = 5
Orphan	1	0

* Numbers and abbreviations are as described in [23].

Rhodopsin family in *Tetraodon *has up to one and half times the number of receptors compared with human (excluding olfactory receptors), whereas about two fold as many GPCR sequences as in fugu and about three fourth of the zebrafish GPCRs [19]. *Tetraodon *also has similar numbers of frizzled receptors as expected in mammals and fish genomes. Some of the gene families in *Tetraodon *like opsins and fish odorant receptors have shown species-specific expansions similar to trace amine receptors in zebrafish [20]. However, taste receptors type 2 (TAS2) and mas related (MRG) receptors seem to be absent in *Tetraodon *like other known fish genomes [19].

**Figure 2:**
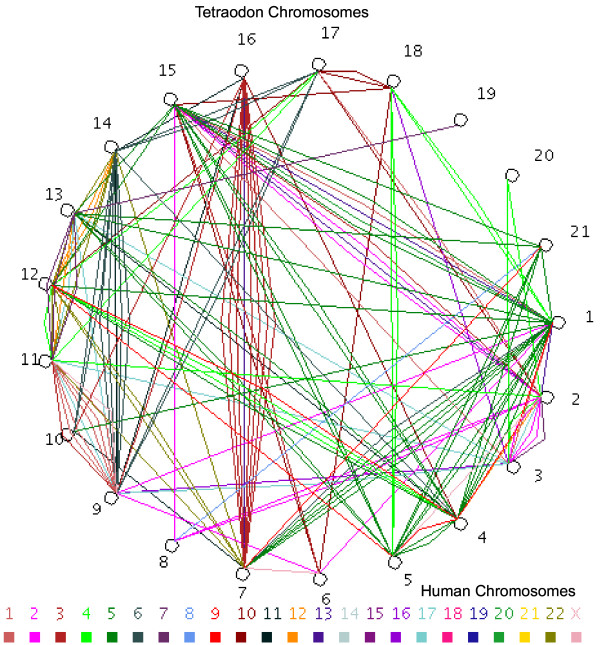
**Global distribution of GPCRs in the *Tetraodon *genome**. The 21 *Tetraodon *chromosomes are presented in a circle and each line joins GPCR paralogs on a given pair of chromosomes. The color of the line indicates the chromosomal location of the corresponding human orthologue.

Analysis of the chromosomal distribution of Tnig-GPCRs show their distribution across all the chromosomes and GPCRs on one chromosome show a greater tendency to have duplicated copies located on another chromosome (Figure 2; shaded in gray in Additional data file 1). Comparative genomic studies of *Tetraodon *and humans show many GPCRs for which there are two copies in *Tetraodon *but one in the human genome. Chromosomal distribution of putative *Tetraodon*-human GPCR orthologous pairs and corresponding Tnig-GPCR paralogs show correspondence between two different chromosomal regions in *Tetraodon *genome to one region in the human genome (Figure 2). This two to one (2:1) association also supports the hypothesis that these genes arose through a large-scale gene duplication event, probably involving whole genome duplication in *Tetraodon *[14,21,22], since almost all *Tetraodon *chromosomes are involved.

**Figure 3:**
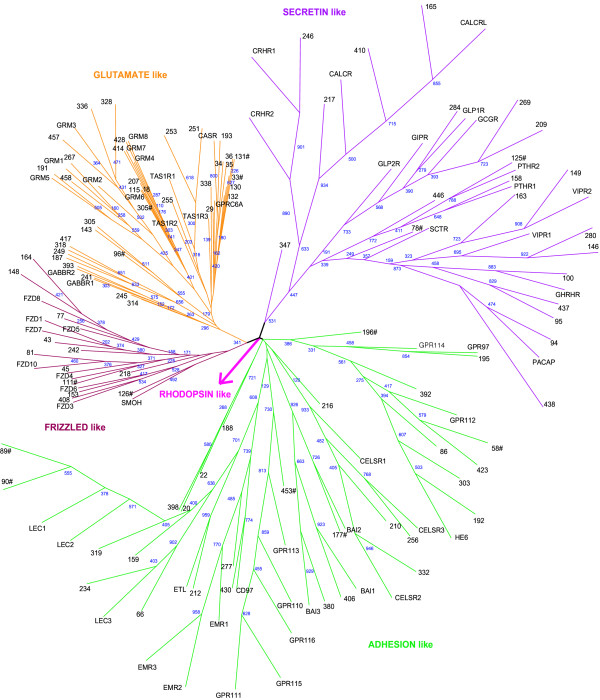
**Phylogenetic relationship between GPCRs in the *Tetraodon *and human genome**. The position of the rhodopsin family was established by including fifty receptors randomly from the rhodopsin family. These branches were removed from the final figure and replaced by an arrow towards rhodopsin family analysis in Additional data file 2. Numbers in black refers to *Tetraodon *GPCRs as per number represented in Additional data file 1. Tnig-GPCRs with unusual lengths of the predicted 7TM domain (under predicted or over predicted TM helices; please see Methods for details), are marked using a '#' symbol.

**Figure 4:**
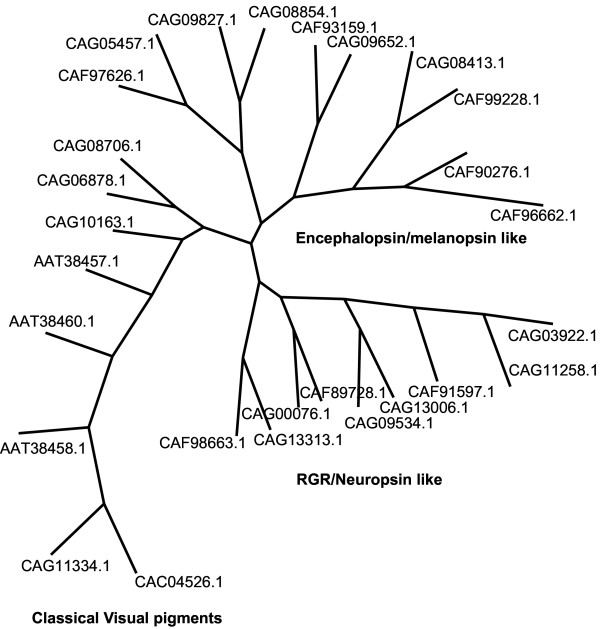
Phylogenetic tree of the *Tetraodon *opsin receptors performed using the neighbor-joining method.

GPCR classification has been proposed by Fredriksson, and Schioth in human and other fully sequenced genomes into five main families; glutamate (G), rhodopsin (R), adhesion (A), frizzled (F) and secretin (S) (GRAFS classification) [19,23,24].*Tetraodon *GPCRs also show five main GRAFS [G with 36 members; R, 368 (see Additional data file 2); A, 29; F, 12 and S, 21] families (Figure 3). It is observed, however, in *Tetraodon *that there were shifts of some of the receptors between the main groups of rhodopsin family [24]. Under the rhodopsin family, there are nine opsin receptor representations in humans, but *T. nigroviridis *displays an expansion where we have identified 27 Tnig-opsin receptors. The phylogenetic analysis divides *Tetraodon *opsins into three branches: classical visual pigments, neuropsin/RGR like, and encephalopsin/melanopsin like (Figure 4). There are at least four copies of genes under each of these branches in *Tetraodon*, but only one orthologous copy each has been identified in human genome, indicating fish specific gene duplications as observed earlier for trace amine receptors in zebrafish [20,25].

**Figure 5:**
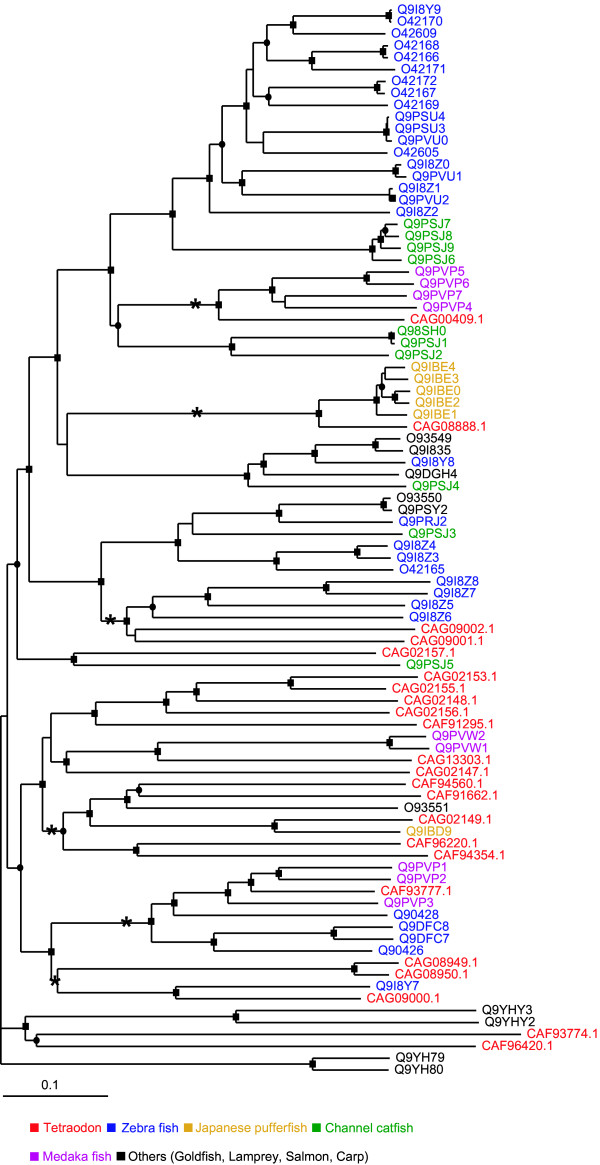
**Phylogenetic tree of the *Tetraodon *and fish odorant receptors**. *Tetraodon *odorant receptors are in red. Bootstrap support is indicated with circle on the relevant branch point for 60 to 80% and a square for 80 to 100%. Branches of possibly orthologous grouping of odorant receptors are marked with an asterisk.

23 candidate odorant receptors (OR) were identified in fish odorant receptor (FOR) subfamily of rhodopsins in *Tetraodon*. These OR genes are found in clusters of 3–4 members in the *Tetraodon *genome, located on different chromosomes. They display higher sequence identity within a cluster suggesting tandem duplication events might be responsible for OR gene family expansion in *Tetraodon *as observed in the genomes of every vertebrate organism investigated earlier, including zebrafish, mice and humans [26]. Phylogenetic analysis of *Tetraodon *ORs with fish odorant receptor subfamily members (mainly zebrafish, channel catfish, Japanese pufferfish, medaka fish, goldfish etc) grouped them into six clusters of orthologues with very high boot strap support (Figure 5). In teleost lineage, different members of FOR subfamily have shown species specific gene expansion. For example, there is a large group of FORs with 18 zebrafish members, 6 catfish members, 4 medaka fish and one each of *Tetraodon *and channel catfish. Another group consists of 12 *Tetraodon *members, 2 medaka fish members and one each of goldfish and Japanese pufferfish (Figure 5). High differences in numbers of OR genes in specific fish reflect creature-specific lifestyle and these receptors are responsible for binding ligands important to a particular species [18-20,25].

**Figure 6:**
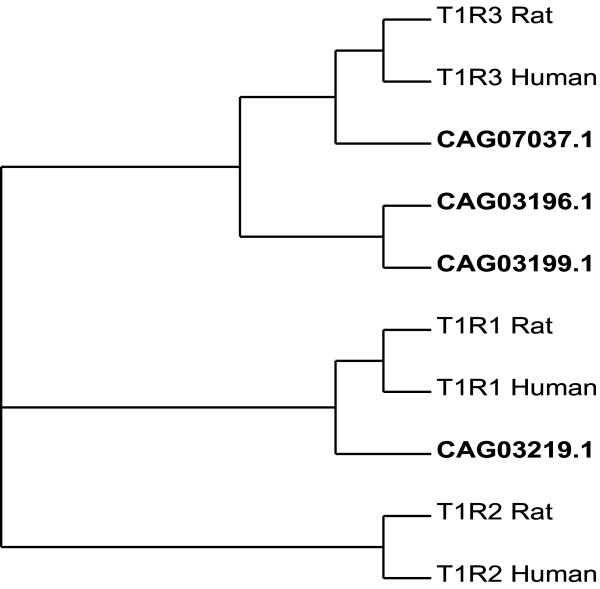
**Phylogenetic tree of the *Tetraodon*, human and rat taste receptors (T1Rs)**. *Tetraodon *T1Rs are represented in bold.

Among the glutamate receptor family, we find four novel members of candidate mammalian type-1 (T1Rs) taste receptors in *Tetraodon *genome (Figure 6). They have been implicated in sweet and umami detection in mammals by forming homo and/or hetero dimers [27,28]. Tnig-taste receptors retain several conserved ligand binding residues when compared to rat mGluR1 metabotropic glutamate receptor [27] (Accession no. P23385; PDB entry no. 1EWK; see Additional data file 3). Phylogenetic analysis of T1R receptors in human, rat and *Tetraodon *reveals two groups of Tnig-taste receptors: with one T1R1-like gene and other with three T1R3-like genes. A putative human GPCR orthologue has been identified for both groups. The presence of T1R family members in the *Tetraodon *genome suggests that the emergence of dimer-forming chemosensory receptors of glutamate family antedate the emergence of land vertebrates.

## Conclusion

We have identified and analyzed repertoire of *Tetraodon *GPCRs and found high level of orthology with human counterparts. The human and *Tetraodon *GPCR sequences are analogous in terms of GPCR subfamilies, but display disproportionate number of receptors at the subfamily level. The teleost genome, with its expanded set of GPCRs, provides an additional and interesting model to study both evolution and function of these receptors. The availability of repertoire of *Tetraodon *GPCRs will facilitate further studies through "functional genomics" and "reverse pharmacological" strategies to match their cognate ligands and to elucidate biological functions. Systematic mutation of *Tetraodon *GPCRs will help to determine their neural, developmental and behavioral roles. They might also yield novel insights into the physiological functions and mutational pathologies of their human homologues in particular and other vertebrate homologues in general.

## Methods

### Identification of Tnig-GPCRs

Sequences of the *Tetraodon nigroviridis *are obtained from NCBI and Genoscope *Tetraodon *Genome Browser [29]. HumanGPCR sequences were identified using GPCRDB [30] (Release 8.1) and based on earlier studies [7,19,23,31]. GPCRs were identified using comprehensive approach (Figure 1) that includes RPS-BLAST [32] (using CDD v2.01 [33]: SMART [34], Pfam [35] and COG Databases; E-value cut-off 10-5), Hmmpfam of HMMER 2.3.2 [36] (using Pfam15; E-value cut-off 0.01) and BLASTP [37] homology comparisons against GPCRDB. Putative GPCR sequences were manually checked for GPCR specific patterns and presence of 7TM domain (at least 70% or more of Pfam 7TM should be aligned with each of the sequence). This is followed by secondary structure (transmembrane helix(TMH)) predictions using one or more methods like HMMTOP [38], SOSUI [39], MEMSTAT [40] and TMHMM2 [41]. A range of 6–8 predicted TM helices acquired maximum coverage (96 percent; please see Additional data file 4 for details) when tested on a dataset of 327 annotated human GPCRs. A similar range was set to recognize acceptable tetraodon protein sequences containing transmembrane domain. Other examples, that either have under predicted or over predicted number of TM helices are earmarked separately ('#' symbol) in the current analysis. Splice variants, polymorphism and duplicates were eliminated by applying 90% sequence identity cut-off using CD-hit [42] and also checked manually. The corresponding genomic DNA sequences were also searched against the EST database at NCBI using BLASTN with a cutoff E-value of 1e^12 ^[20]. We could not obtain any *Tetraodon nigroviridis *EST hits, as there were few or no *Tetraodon nigroviridis *EST sequences available in the database.

### Ortholog identification

Two genes, A from genome GA and B from GB, were considered orthologs if B is the best match of gene A in GB and A is the best match of B in GA using BLASTP [14].

### Phylogenetic analysis

Preliminary phylogenetic analysis [43] was performed using neighbor joining method with fewer number of bootstrap replicas and no randomization of sequence order. This was sufficient to separate GPCR sequences into rhodopsin like receptors and non rhodopsin like receptors. Rhodopsin like receptor and non-rhodopsin like receptor sequence datasets (separately full length and 7TM domain only), along with respective human GPCRs, were separately randomized twenty times with regard to sequence input order using a script called RandSeq (available upon request). These twenty datasets of different sequence order were aligned using clustalX 1.83 [44] using multiple sequence alignment parameters with protein weight matrix BLOSUM series, gap opening penalty 10.0 and gap extension penalty 0.05 and delay divergence of 35 percent. To obtain unrooted trees, each alignment was bootstrapped 50 times and neighbor joining method (NEIGHBOR; Phylip package [45]) was employed to obtain tree topology using distance matrices obtained from alignments by PRODIST [45]. Consensus tree was obtained from 1000 neighbor trees using CONSENSE [45]. Only 500 boot strap replicas were used for rhodopsin like receptors due to limitations in the CONSENSE program and the trees were generated using Treeview [46]. Maximum-likelihood tree of non-rhodopsin like receptors were also inferred from the alignment using TREE-PUZZLE [47]. 10,000 quartet-puzzling steps were performed to obtain support values (reliability) for each internal branch.

## Authors' contributions

M.R.P.R. has carried out the work and has written the first draft of the manuscript. R.S. had initiated the idea and was involved in useful discussions and drafting of the final manuscript.

## Supplementary Material

Additional data file 1 ***Tetraodon nigroviridis *G protein-coupled receptors** Click here for file

Additional data file 2 **Phylogenetic relationship between GPCRs in the *Tetraodon *and human rhodopsin family **Eighty receptors from almost all subfamilies of human rhodopsin family were randomly included along with all members of rhodopsin family of *Tetraodon *to construct the phylogenetic tree. Numbers in black refers to *Tetraodon *GPCRs as per number represented in Additional data file 1. Click here for file

Additional data file 3 **Sequence alignment of the *Tetraodon*, human and rat taste receptors (T1Rs) **T1Rs of *Tetraodon*, human and rat are aligned with the rat mGluR1 metabotropic glutamate receptor (Accession no. P23385). Ligand binding residues of mGluR1 are highlighted in red. The C-terminus is not shown. Potential transmembrane segments are indicated using arrows. Click here for file

Additional data file 4 **Transmembrane Helix (TMH) prediction of human GPCRs by different TMH prediction programs (HMMTOP, SOSUI, TMHMM and MEMSAT) **A dataset of 327 annotated human GPCRs are predicted for Transmembrane Helices (TMH) by HMMTOP, SOSUI, TMHMM and MEMSAT. A range of 6–8 predicted TM helices acquired maximum coverage to predict 7TM domain region. Click here for file
