# Supplementary material for: Genome wide survey of G protein-coupled receptors in Tetraodon nigroviridis
Source: BMC Evol Biol. 2005 Jul 15;5:41. doi: 10.1186/1471-2148-5-41 (PMC1187884; doi:10.1186/1471-2148-5-41)
Supplement: Additional data file 3 — Sequence alignment of the Tetraodon, human and rat taste receptors (T1Rs) T1Rs of Tetraodon, human and rat are aligned with the rat mGluR1 metabotropic glutamate receptor (Accession no. P23385). Ligand binding residues of mGluR1 are highlighted in red. The C-terminus is not shown. Potential transmembrane segments are indicated using arrows. [file 1471-2148-5-41-S3.pdf]

```
hT1R1         -------MLLCTARLVGLQLLISCCWAFACHSTESS---PDFTLPGDYLLAGLFPLHSGCLQVRHR----
rT1R1         -------MLFWAAHLL-LSLQLVYCWAFSCQRTESS---PGFSLPGDFLLAGLFSLHGDCLQVRHR----
CAG03219.1    -------MHLQTGTFVAILSTTNLVLQLVRGELDRSSHGQGMQLHGNFSIAGFFPLHYGYEPDGRLP---
rT1R2         --------MGPQARTLCLLSLLLHVLPKPGKLVENSD----FHLAGDYLLGGLFTLHANVKSISHLS---
hT1R2         --------MGPRAKTICSLFFLLWVLAEP---AENSD----FYLPGDYLLGGLFSLHANMKGIVHLN---
rT1R3         ---------MPGLAILGLSLAAFLELGMGSSLCLSQQ----FKAQGDYILGGLFPLGTTEEATLNQR---
hT1R3         ---------MLGPAVLGLSLWALLHPGTGAPLCLSQQ----LRMKGDYVLGGLFPLGEAEEAGLRSR---
CAG07037.1    --------MAVPPTLLVFLLVCKLTCSTPAWFQNISTN--LFNLPGDIMLGGLFPINQLTSNLSQRT---
CAG03196.1    MSEGDSVGESIHGKPSVVSAFFTRIGQIYQSWLDKSTPFYAVRWAATLLLTAVYMIRVYLLQGWYIVTYA
CAG03199.1    ----------MGRSLACLCLLGLVLYPLARGTVPASE----FRLEGDYLLGGLFEIHYDTFPTPHDR---
mGluR1        ---MVRLLLIFFPMIFLEMSILPRMPDRKVLLAGASSQRSVARMDGDVIIGALFSVHHQPPAEKVPE---

hT1R1         ---------------PEVTLCDRSCSFNEHGYHLFQAMRLGVEEINNST---ALLPNITLG--YQLYDVC
rT1R1         ---------------PLVTSCDRPDSFNGHGYHLFQAMRFTVEEINNSS---ALLPNITLG--YELYDVC
CAG03219.1    ----------------ALALCKDGD-LNKHGFHLLHAMKLAVEEINSNTGAQSLLPGVMLG--YQMYDTC
rT1R2         --------------YLQVPKCN-EFTMKVLGYNLMQAMRFAVEEINNCS---SLLPGVLLG--YEMVDVC
hT1R2         --------------FLQVPMCK-EYEVKVIGYNLMQAMRFAVEEINNDS---SLLPGVLLG--YEIVDVC
rT1R3         ----------------TQPNGILCTRFSPLGLFLAMAMKMAVEEINNGS---ALLPGLRLG--YDLFDTC
hT1R3         ----------------TRPSSPVCTRFSSNGLLWALAMKMAVEEINNKS---DLLPGLRLG--YDLFDTC
CAG07037.1    -----------------EPDQITCDRIDSFGLGMAIVMKYAVDEINANQ---FLLPGIRLG--YEIHDTC
CAG03196.1    LGIYHLNLFIAFLSPKVDPSLLDEDEGPALPTKQNEEFRPFIRRLPEFKFWHSATKGIVIAMVCTFFEVF
CAG03199.1    ----------------PESLDCSSKTLFLSNYRRFQLMRFAVAEINNST---DLLPNVTLG--YEVFDHC
mGluR1        --------------------RKCGEIREQYGIQRVEAMFHTLDKINADP---VLLPNITLG--SEIRDSC

hT1R1         SDSAN-VYATLRVLSLPGQHHIELQGDLLHYSP-------------------------------TVLAVI
rT1R1         SESAN-VYATLRVLALQGPRHIEIQKDLRNHSS-------------------------------KVVAFI
CAG03219.1    SESAS-ILASLDVLDYWSSSASGTEPDFSTSQR--------------------------------PLAVI
rT1R2         YLSNN-IHPGLYFLAQD-DDLLPILKDYSQYMP-------------------------------HVVAVI
hT1R2         YISNN-VQPVLYFLAHE-DNLLPIQEDYSNYIS-------------------------------RVVAVI
rT1R3         SEPVVTMKPSLMFMAKVGSQSIAAYCNYTQYQP-------------------------------RVLAVI
hT1R3         SEPVVAMKPSLMFLAKAGSRDIAAYCNYTQYQP-------------------------------RVLAVI
CAG07037.1    RQSAVIIRPTIYYLRAKHDNNLIAQCNYTNYET-------------------------------RISAVI
CAG03196.1    NVPVFWPILVMYFIMLFCITMKRQIKHMIKYRYLPFTHGKRTYKGKDETGKPLHLNEFFIRMLKSKLSTL
CAG03199.1    SDTRCFAGIFKLLSVDNVVQPWNVPGKKVS----------------------------------KVVAVV
mGluR1        WHSSVALEQSIEFIRDSLISIRDEKDGLNRCLPDGQTL-------------------PPGRTKKPIAGVI

hT1R1         GPDS-----------------------------------------TNRAATTAALLSPFLVPMISYAASS
rT1R1         GPDN-----------------------------------------TDHAVTTAALLGPFLMPLVSYEASS
CAG03219.1    GPDS-----------------------------------------SSKSFTPATLLGAYLVPQVSYEASN
rT1R2         GPDN-----------------------------------------SESAITVSNILSHFLIPQITYSAIS
hT1R2         GPDN-----------------------------------------SESVMTVANFLSLFLLPQITYSAIS
rT1R3         GPHS-----------------------------------------SELALITGKFFSFFLMPQVSYSASM
hT1R3         GPHS-----------------------------------------SELAMVTGKFFSFFLMPQVSYGASM
CAG07037.1    GPNN-----------------------------------------SELVSVIGKLLGFFLMPQISYGATS
CAG03196.1    NPSSQSFLLPNYRRFQLMRFSMEEINNSSDLLPGVSLGYEIFDICSDLQSFPGVLKQISVNGSVSYGASS
CAG03199.1    GLFT-----------------------------------------STFTLTVAPLFMMDFIPMISYGSSS
mGluR1        GPGS-----------------------------------------SSVAIQVQNLLQLFDIPQIAYSATS

hT1R1         ETLSVKRQYPSFLRTIPNDKYQVETMVLLLQKFGWTWISLVGSSDDYGQLGVQALENQATGQ-GICIAFK
rT1R1         VVLSAKRKFPSFLRTVPSDRHQVEVMVQLLQSFGWVWISLIGSYGDYGQLGVQALEELAVPR-GICVAFK
CAG03219.1    EMLSNKIFYPAFFRTIPSDKNQVAAMIQLLVRFNWTWVALLGSDNSYGLEGMRSLSQQAPDH-HICIAYQ
rT1R2         DKLRDKRHFPSMLRTVPSATHHIEAMVQLMVHFQWNWIVVLVSDDDYGRENSHLLSQRLTKTSDICIAFQ
hT1R2         DELRDKVRFPALLRTTPSADHHVEAMVQLMLHFRWNWIIVLVSSDTYGRDNGQLLGERVARR-DICIAFQ
rT1R3         DRLSDRETFPSFFRTVPSDRVQLQAVVTLLQNFSWNWVAALGSDDDYGREGLSIFSGLANSR-GICIAHE
hT1R3         ELLSARETFPSFFRTVPSDRVQLTAAAELLQEFGWNWVAALGSDDEYGRQGLSIFSALAAAR-GICIAHE
CAG07037.1    DKFSDNILYPSFFRTVPSDKGQVEAMVLLLLEFNWNWVAVVGSDEEYGQRGVQDFSKVAANR-SICVAYQ
CAG03196.1    SVFSSNENFPSFLRTVHPNKEVINVIIRILQHFNWRWAAFLNSDNDFGIDGRNLFIQGIKDT-DICLAYT
CAG03199.1    SILSEKVKFPSFLRTVHSSQEVIQVIVKLLQYFKWHWVAFLYSNDDYGANAQKLFIEKIKNT-DICLAYT
mGluR1        IDLSDKTLYKYFLRVVPSDTLQARAMLDIVKRYNWTYVSAVHTEGNYGESGMDAFKELAAQE-GLCIAHS
```

```
hT1R1        DIMPFSAQVG------DERMQCLMRHLAQ--AGATVVVVFSSRQLARVFFESVVLTNLT-GKVWVASEAW
rT1R1        DIVPFSARVG------DPRMQSMMQHLAQ--ARTTVVVVFSNRHLARVFFRSVVLANLT-GKVWVASEDW
CAG03219.1   GVIPEYTRDT------VALMRNIVDGILM--TKVTTIVVFSSKSKLSKFMPFVIERNVT-EKVWIGTEDW
rT1R2        EVLPIPESSQVMRSEEQRQLDNILDKLRR--TSARVVVVFSPELSLYSFFHEVLRWNFT-GFVWIASESW
hT1R2        ETLPTLQPNQNMTSEERQRLVTIVDKLQQ--STARVVVVFSPDLTLYHFFNEVLRQNFT-GAVWIASESW
rT1R3        GLVPQHDTSG----QQLGKVVDVLRQVNQ--SKVQVVVLFASARAVYSLFSYSILHDLS-PKVWVASESW
hT1R3        GLVPLPRADD----SRLGKVQDVLHQVNQ--SSVQVVLLFASVHAAHALFNYSISSRLS-PKVWVASEAW
CAG07037.1   GLIPVYTDPE-------PVVKTIISNINS--TKANVVIVFSLPNQAEIFFQEVIRTELK--GVWIGSTAW
CAG03196.1   ENLNMLTDFT----------QTFQQINL--QKINVIIVFAPKMFVEALINSAIKLNIS-DKVWIADDGW
CAG03199.1   HDLVSSDFLS-----------IFQWIKS--QKIVVIVVMAPEWSAKPLVKSAIQHNVT-DKVWIAGEAW
mGluR1       DKIYSNAGEK--------SFDRLLRKLRERLPKARVVVCFCEGMTVRGLLSAMRRLGVVGEFSLIGSDGW

hT1R1        ALSRHITGVPGIQRIGMVLGVAIQKRAVPG-LKAFEEAYARADKKA----PRPCHK--------GSWCSS
rT1R1        AISTYITSVTGIQGIGTVLGVAVQQRQVPG-LKEFEESYVRAVTAA----PSACPE--------GSWCST
CAG03219.1   SQSSLIAGIPGIHTIGTVIGVAIKYTVIPGFVERLAEASLQRSNEE-----NASTV----------TVNP
rT1R2        AIDPVLHNLTELRHTGTFLGVTIQRVSIPG-FSQFRVRRDKPGYPV----PNTTNL--------RTTCN-
hT1R2        AIDPVLHNLTELGHLGTFLGITIQSVPIPG-FSEFREWGPQAGPPP----LSRTSQ--------SYTCN-
rT1R3        LTSDLVMTLPNIARVGTVLGFLQRGALLPEFSHYVETRLALAADP-----TFCASL---KAELDLEERVM
hT1R3        LTSDLVMGLPGMAQMGTVLGFLQRGAQLHEFPQYVKTHLALATDP-----AFCSALG--EREQGLEEDVV
CAG07037.1   TISERVFLLPNLHTVGTILGFAFTTQSLDLLRAYTHELLTKVSEEH-AHTPPPVEN----------LNDY
CAG03196.1   SLNKNLPKKEGIRRIGTVIGVSQPVVTIPAFDDFIFSSKSQKQCE--------------------EREE
CAG03199.1   SLNKELPKEKGIKHIGTVIGVAQLYLNIPRFQDFIYSVKKQNECES---------------------AE
mGluR1       ADRDEVIEGYEVEANGGIT-IKLQSPEVRSFDDYFLKLRLDTNTRNP-WFPEFWQHRFQCRLPGHLLENP

hT1R1        NQLCRECQAFMAHTMPKLKAFS------MSSAYNAYRAVYAVAHGLHQLLG--CASGA--CS---RGRVY
rT1R1        NQLCRECHTFTTRNMPTLGAFS------MSAAYRVYEAVYAVAHGLHQLLG--CTSEI--CS---RGPVY
CAG03219.1   NNTCLQSRELYSLAEMNLPLDN----YDIASAVNVYKAVYAVAHALHQLLR--CGSGE--CQ---RKRVY
rT1R2        -QDCDACLNTTKSFNNILILSG------ERVVYSVYSAVYAVAHALHRLLG--CNRVR--CT---KQKVY
hT1R2        -QECDNCLNATLSFNTILRLSG------ERVVYSVYSAVYAVAHALHSLLG--CDKST--CT---KRVVY
rT1R3        GPRCSQCDYIMLQNLSSGLMQNLSAGQLHHQIFATYAAVYSVAQALHNTLQ--CNVSH--CH--TSEPVQ
hT1R3        GQRCPQCDCITLQNVSAGLN--------HHQTFSVYAAVYSVAQALHNTLQ--CNASG--CP--AQDPVK
CAG07037.1   SNSCPHCWNLSPANISLDAEVA-----IQRSAFRVYAAIYSVAQALHNLLQ--CNSTA--CMKTSEVQIY
CAG03196.1   QPFCNQACKCGYLSAEEITSAD------PTFSFPVYSAVYAIAHALHKVLQ--CGAGS--CNRS--VTVH
CAG03199.1   QQFCNRVCKCSNVSAEDTLAVD------PSYAFSVYSAVYAAAHALHNVLN--CGTGK--CDSG--VSVS
mGluR1       NFKKVCTGNESLEENYVQ----------DSKMGFVINAIYAMAHGLQNMHHALCPGHVGLCDAMK--PID

hT1R1        PWQLLEQIHKVHFL-LHKDTVAFNDNRDP-LSSYNIIAWDWNGPKWTFTVLGSSTWSPVQLNINETKIQW
rT1R1        PWQLLQQIYKVNFL-LHENTVAFDDNGDT-LGYYDIIAWDWNGPEWTFEIIGSASLSPVHLDINKTKIQW
CAG03219.1   PWELLSRLKQVQFP-MANASVYFDSNGDP-PTGYDIISWVWRGAKWSVRVVGSFSPTPISLTVDADAVEW
rT1R2        PWQLLREIWHVNFT-LLGNRLFFDQQGDM-PMLLDIIQWQWDLSQNPFQSIASYSPTSKRLTY-INNVSW
hT1R2        PWQLLEEIWKVNFT-LLDHQIFFDPQGDV-ALHLEIVQWQWDRSQNPFQSVASYYPLQRQLKN-IQDISW
rT1R3        PWQLLENMYNMSFR-ARDLTLQFDAKGSV-DMEYDLKMWVWQSPTPVLHTVGTFNGT---LQLQHSKMYW
hT1R3        PWQLLENMYNLTFH-VGGLPLRFDSSGNV-DMEYDLKLWVWQGSVPRLHDVGRFNGS---LRTERLKIRW
CAG07037.1   PWKLLKALRNTTVD-INGTMLKFDRNGNP-NIGYSVVELILKNSSLEFLEVGSFNKI---LDIKKSLFKW
CAG03196.1   PHMVLAELKKSNFT-LLNTTIRFNEYGDPKFGSYSIVFWNDKGFPEEVGFFHYFPAT--KFYINGTKIQW
CAG03199.1   PPALLAELKRSNFT-LLNQTIHFNEDGDPLFGSYAVHFWNRSGAAENFGICSFYPSIQ--IFINESKIQW
mGluR1       GRKLLDFLIKSSFVGVSGEEVWFDEKGDA-PGRYDIMNLQYTEAN-RYDYVHVGTWHEGVLNIDDYKIQM

hT1R1        HGK--DNQVPKSVCSSDCLEGHQRVV-TGFHHCCFECVPCGAGTFLNK-SDLYRCQPCGKEEWAPEGSQT
rT1R1        HGK--NNQVPVSVCTTDCLAGHHRVV-VGSHHCCFECVPCEAGTFLNM-SELHICQPCGTEEWAPKESTT
CAG03219.1   HISGDSRSVPQSICSPPCEPGHKRLL-TGQHECCFDCQACAEATFLNE-SDPTTCQTCLPEEWAPKSSQR
rT1R2        YTP--NNTVPVSMCSKSCQPGQMKKS-VGLHPCCFECLDCMPGTYLNRSADEFNCLSCPGSMWSYKNDIT
hT1R2        HTV--NNTIPMSMCSKRCQSGQKKKP-VGIHVCCFECIDCLPGTFLNHTEDEYECQACPNNEWSYQSETS
rT1R3        PG----NQVPVSQCSRQCKDGQVRRV-KGFHSCCYDCVDCKAGSYRKH-PDDFTCTPCGKDQWSPEKSTT
hT1R3        HTS--DNQKPVSRCSRQCQEGQVRRV-KGFHSCCYDCVDCEAGSYRQN-PDDIACTFCGQDEWSPERSTR
CAG07037.1   HTE--NSEVPESTCSAACGEGQVHRV-KGFHSCCFDCIDCLPGTYQRE-KDDIQCTPCPPRQWSLKRSTR
CAG03196.1   FQN---GEVPTSLCSTECLEGYIKKL-NGIHKCCFTCEVCPMGTFVNSTADPYSCLPCEKHQWSTAGSTS
CAG03199.1   HSK----EVPVSQCSKECEEGNAKRQ-EGIHRCCFTCEICPNGTYVNITADPYSCLPCEKHQWSTAGSTS
```

```
mGluR1      NK----SGMVRSVCSEPCLKGQIKVIRKGEVSCCWICTACKENEFVQD---EFTCRACDLGWWPNAELTG
                                      TM-I                                  TM-II

hT1R1       CFPRTVVFLALREHTSWVLLAANTLLLLLLLGTAGLFAWHLDTPVVRSAGGRLCFLMLGSLAAGSGSLYG
rT1R1       CFPRTVEFLAWHEPISLVLIAANTLLLLLLVGTAGLFAWHFHTPVVRSAGGRLCFLMLGSLVAGSCSFYS
CAG03219.1  CLKRTRLLLEWDHPMSVALLFFLVCCLLMTSSSAVILLLNINTPVAKSAGGRTCLLMLAALTAAAMSSLC
rT1R2       CFQRRPTFLEWHEVPTIVVAILAALGFFSTLAILFIFWRHFQTPMVRSAGGPMCFLMLVPLLLAFGMVPV
hT1R2       CFKRQLVFLEWHEAPTIAVALLAALGFLSTLAILVIFWRHFQTPIVRSAGGPMCFLMLTLLLVAYMVVPV
rT1R3       CLPRRPKFLAWGEPAVLSLLLLLCLVLGLTLAALGLFVHYWDSPLVQASGGSLFCFGLICLGLFCLSVLL
hT1R3       CFRRRSRFLAWGEPAVLLLLLLLSLALGLVLAALGLFVHHRDSPLVQASGGPLACFGLVCLGLVCLSVLL
CAG07037.1  CTDPTYDYLSWDTPEALLLTLAIVLVVLLKGAVVVLFLKHRGTVLVAASGGTLSFVVLLGLMGACLSLLL
CAG03196.1  CLPRTVEWVPFTDPAAVVILAGACLLVALTAAMCVLFAVNYNTPVVRSAGGPMCFLILGCLSLCSISVFF
CAG03199.1  CLPRTVEWVPFTDPAAVVILAGACLLVALTAAMCVLFAVNYNTPVVRSAGGPMCFLILGCLSLCSISVFF
mGluR1      CEPIPVRYLEWSDIESIIAIAFSCLGILVTLFVTLIFVLYRDTPVVKSSSRELCYIILAGIFLGYVCPFT
                                      TM-III                                TM-IV

hT1R1       FFGEPTRPACLLRQALFALGFTIFLSCLTVRSFQLIIIFKFSTKVPTFYHAWVQNHGAGLFVMISSAAQL
rT1R1       FFGEPTVPACLLRQPLFSLGFAIFLSCLTIRSFQLVIIFKFSTKVPTFYRTWAQNHGAGLFVIVSSTVHL
CAG03219.1  HFGQPSPLACMLKQPLFTFSFTVCLACIAVRSLQVVCIFKFASRLPPAYDRWAKNHGPEATIFIASAAIL
rT1R2       YVGPPTVFSCFCRQAFFTVCFSICLSCITVRSFQIVCVFKMARRLPSAYSFWMRYHGPYVFVAFITAIKV
hT1R2       YVGPPKVSTCLCRQALFPLCFTICISCIAVRSFQIVCAFKMASRFPRAYSYWVRYQGPYVSMAFITVLKM
rT1R3       FPGRPRSASCLAQQPMAHLPLTGCLSTLFLQAAEIFVESELPLSWANWLCSYLRGPWAWLVVLLATLVEA
hT1R3       FPGQPSPARCLAQQPLSHLPLTGCLSTLFLQAAEIFVESELPLSWADRLSGCLRGPWAWLVVLLAMLVEV
CAG07037.1  FLGQPGDTVCRLQLPLVSIFQTVPLCIIMSISLQIFFVSEFP-TLAASYLHVLRGPGTWLLLLICCAVQA
CAG03196.1  YFERPTEAFCILRFMPFLLFYAVCLACFAVRSFQIVIIFKIAAKFPRVHSWWMKYHGQWLVISMTFVLQA
CAG03199.1  YFERPTEAFCILRFMPFLLFYAVCLACFAVRSFQIVIIFKIAAKFPRVHSWWMKYHGQWLVISVAFVLQA
mGluR1      LIAKPTTTSCYLQRLLVGLSSAMCYSALVTKTNRIARILAGSKKKICTRKPRFMSAWAQVIIASILISVQ
                                                        TM-V

hT1R1       LICLTWLVVWT-PLPA-REYQRFPHLVMLECTETNSLGFILAFLYNGLLSISAFACSYLGKDLPENYNEA
rT1R1       LICLTWLVMWT-PRPT-REYQRFPHLVILECTEVNSVGFLLAFTHNILLSISTFVCSYLGKELPENYNEA
CAG03219.1  CVSVLRVAVGP-PQPS-QDLDFYTDSIVLECSNTLSPGSFVELCYVSLLSAVCFVFSYMGKDLPANYNEA
rT1R2       ALVVGNMLATT-INPIGRTDPDDPNIMILSCHPNYRNGLLFNTSMDLLLSVLGFSFAYMGKELPTNYNEA
hT1R2       VIVVIGMLATG-LSPTTRTDPDDPKITIVSCNPNYRNSLLFNTSLDLLLSVVGFSFAYMGKELPTNYNEA
rT1R3       ALCAWYLMAFP-PEVV-TDWQVLPTEVLEHCRMRSWVSLGLVHITNAVLAFLCFLGTFLVQSQPGRYNRA
hT1R3       ALCTWYLVAFP-PEVV-TDWHMLPTEALVHCRTRSWVSFGLAHATNATLAFLCFLGTFLVRSQPGRYNRA
CAG07037.1  GICGWFVQEGPSLSEYLANRRIDFVRSFLACPVSSLSGFGLMQGLITVMALISFMCTFMATKPLHQYNLA
CAG03196.1  VVIVIGFSSDP--PLPYSNFLSYPDKIILGCDVN-LNMASTSFFLLLLCILCFTFSYMGKDLPKNYNEA
CAG03199.1  VLIVIAFSSDP--PLPYSDFLSYPDKIILGCDVN-LNMASTSFFFASIVVHPLLYFLLHGERPPEELQRG
mGluR1      LTLVVTLIIME-PPMPILSYPSIKEVYLICNTSN--LGVVAPVGYNGLLIMSCTYYAFKTRNVPANFNEA
                      TM-VI                         TM-VII

hT1R1       KCVTFSLLFNFVSWIAFFTTASVYDGKYLPAANMMAGLSSLSSGFGGYFLPKCYVILCRPDLNSTEHFQA
rT1R1       KCVTFSLLLNFVSWIAFFTMASIYQGSYLPAVNVLAGLTTLSGGFSGYFLPKCYVILCRPELNNTEHFQA
CAG03219.1  KCVTFSLMVYMISWISFFTVYLISRGPFTMAAYVCATLVSVLAFFGGYFLPKIYIIVLKPHMNTTAHFQN
rT1R2       KFITLSMTFSFTSSISLCTFMSVHDGVLVTIMDLLVTVLNFLAIGLGYFGPKCYMILFYPERNTSAYFNS
hT1R2       KFITLSMTFYFTSSVSLCTFMSAYSGVLVTIVDLLVTVLNLLAISLGYFGPKCYMILFYPERNTPAYFNS
rT1R3       RGLTFAMLAYFIIWVSFVPLLANVQVAYQPAVQMGAILFCALGILATFHLPKCYVLLWLPELNTQEFFLG
hT1R3       RGLTFAMLAYFITWVSFVPLLANVQVVLRPAVQMGALLLCVLGILAAFHLPRCYLLMRQPGLNTPEFFLG
CAG07037.1  RDITFSSLIYCIIWVTFIPIYVGLEEKFKSIVYVSFILTSNLGMVAMYYIPKCYLLLRKPELNTADHF--
CAG03196.1  KAITFCLLLLILTWIIFATAFMLYHGKYIHTLNALAVLSSAYCFLLWYFLPKCYIIIFQPHKNTQKYFQG
CAG03199.1  QSHNLLLASADPHLDHICHRDHALPRQIHPHPQRPGRALQRLLLSPVVFPPQMLHYHLSAPQEHPEVLPG
mGluR1      KYIAFTMYTTCIIWLAFVPIYFGSN--YKIITTCFAVSLSVTVALGCMFTPKMYIIIAKPERNVRSAFTT
```
